# Efficient separation of strontium radionuclides from high-salinity wastewater by zeolite 4A synthesized from Bayer process liquids

**DOI:** 10.1038/s41598-021-81255-y

**Published:** 2021-01-18

**Authors:** Ivana Smičiklas, Ivana Coha, Mihajlo Jović, Marijana Nodilo, Marija Šljivić-Ivanović, Slavko Smiljanić, Željko Grahek

**Affiliations:** 1 “VINČA” Institute of Nuclear Sciences, National Institute of the Republic of Serbia, University of Belgrade, Mike Petrovića Alasa 12-14, 11001 Belgrade, Serbia; 2 Ruđer Bošković Institute, Bijenička cesta 54, 10000 Zagreb, Croatia; 3 University of East Sarajevo, Faculty of Technology , Karakaj 34A, 75400 Zvornik, Republic of Srpska Bosnia and Herzegovina

**Keywords:** Environmental sciences, Chemistry

## Abstract

The efficient, selective, and economical sorbents for the removal of Sr radionuclides are largely needed for the decontamination of effluents with high salinity. In this study, the removal of Sr was investigated using the zeolite produced from the Bayer process liquids. Based on the XRD, SEM/EDS analysis, the product was pure and highly crystalline zeolite 4A (Z4A). Removal of Sr was fast (5 min for 100% removal at 8.80 mg/L), with high maximum sorption capacity (252.5 mg/L), and independent on the initial pH in the range 3.5–9.0. Specific sorption of Sr by protonated groups on the Z4A surface was operating in addition to ion-exchange with Na ions. The selectivity of Z4A decreased in the order Sr > Ca > K > Mg > Na. 84% of Sr was separated from seawater within 5 min, at the Z4A dose of 5 g/L, while efficiency increased to 99% using the dose of 20 g/L. Desorption of radioisotope ^89^Sr from seawater/Z4A solid residue was very low in deionized water (0.1–0.2%) and groundwater (0.7%) during 60 days of leaching. Z4A is a cost-effective, selective, and high-capacity medium for Sr removal, which provides high stability of retained radionuclides.

## Introduction

Liquid radioactive waste (LRW) streams from different sources display a range of radioactive substances with different types of radioactive decay, half-lives (*t*_1/2_), activity concentrations, and chemical properties^1^, but also a variety of inactive constituents^2^. The physicochemical characteristics and the volume of LRW play a critical role in the selection of the treatment process, moreover, they can be more restrictive factors than the radiological properties^3^. If general chemical and physical processes^4,5^ do not provide a sufficient decontamination factor for certain radionuclide, specific treatment steps have to be developed^3^.

Radioactive isotope ^90^Sr is an example of a contaminant that is difficult to separate from the aqueous medium using conventional methods^6^. ^90^Sr is a high yield by-product of uranium fission at nuclear reactors, which undergoes the β-decay with the half-life of 28.8 years. The separation of radiostrontium from the LRW with high salt content is a demanding task^7^, particularly in the presence of seawater (SW)^8^. In the matrix of SW, the mean total Sr concentration is 8.0 mg/L, while the concentration of other cations is far higher (~ 10,000 mg/L Na, 1200 mg/L Mg, 400 mg/L Ca and 400 mg/L K)^9^.

From the aspect of radioecology, ^90^Sr is the most important long-lived anthropogenic radionuclide in the marine environment, principally due to nuclear weapon testing, operation, and accidents of nuclear facilities^10^. Examples of LRW with seawater come from power reactors of civil and navy vessels^11^ and arise following the nuclear accidents like the one at the Fukushima Daiichi nuclear power plant (NPP) where the seawater was initially pumped into the reactors to maintain cooling during the emergency^12^.

For the liquid waste with more than 50% SW, the application of selective sorbents was found necessary^13^. The survey of the latest scientific literature demonstrates the intensified efforts to develop Sr-selective sorbents/ion-exchangers for application in SW. Materials tested in SW encompass inorganic, organic, and composite materials: BaSiO_3_ and BaMnO_4_^13^, Ba-titanate^14^, Ba-impregnated 4A zeolite^15^, macroporous LTA-monoliths^16^ apatite-based, and non-apatite phosphates^17,18^, granulated Na-birnessite^19^, alginate microsphere^20^, MnO_2_-alginate and zeolite-alginate composites^21,22^, magnetic zeolite nanocomposites^23^, and composite magnetic nanoparticles derived from industrial sludge^24^.

Regarding both the selectivity towards Sr and the sorption capacity, all novel materials display advantages over the conventional sorbents and ion-exchange resins, however, the most promising were the ones displaying either the chemical reactivity or ion-sieving effects. The efficacy of the sorption-reagent materials, including BaSiO_3_, BaMnO_4_^13^, Ba-titanate^14^, Ba-impregnated 4A zeolite^15^, is enhanced as a result of their chemical reaction with the anionic components of LRW (SO_4_^2−^), and co-precipitation of Sr with insoluble barium-sulfate. On the other hand, granulated Na-birnessite^19^, a low crystallized layered modification of manganese oxide, exhibited structural benefits for efficient Sr removal in the presence of chemically close elements. The similar effects are operating using synthetic zeolites of LTA type (Linde Type A)^25^. This specific zeolite has lower Si/Al ratio compared to natural zeolites^26^, and the excess of Al^3+^ ions in the crystal lattice provides higher permanent negative charge and higher cation-exchange capacity (CEC). In addition, the specific crystal structure, the distribution of the exchange sites, and their accessibility to a particular cation play significant roles in cation separation^27^. Building units of the sodium form of zeolite 4A are sodalite cages connected by four-membered rings forming a three-dimensional network. The central cavities of these cages having 11.4 Å in diameter are interconnected by eight-ring openings with a 4.1 Å aperture, forming the structure with a high void volume fraction^26^. So far, zeolite 4A samples, prepared from pure chemicals, generally displayed high capacities and the selectivity for Sr^28–31^. However, as the production techniques should be economically sound for obtaining amounts needed in practice^32^, the synthesis of zeolites from readily available and waste materials is encouraged^33–36^. The detail characterization of products obtained from different sources and via different preparation routes is needed, as these factors affect chemical, structural, and surface properties of zeolite 4A^27^.

In this study, the Sr sorption potential of a synthetic zeolite 4A produced from economical sources was assessed, with emphasis on the process efficiency in high saline solutions. The sample of zeolite was supplied from the Alumina plant (Zvornik Alumina Refinery, Bosnia and Herzegovina), which produces over 150,000 tons of zeolite annually using the Alumina refinery products. The main raw materials are: (i) aluminate containing liquid from the Bayer process (Na_2_O—150 g/L; Al_2_O_3_—135 g/L; ρ—1.28 g/mL), (ii) water glass prepared in the factory (Na_2_O—160 g/L; SiO_2_—380 g/L; ρ—1.42 g/mL), and (iii) a portion of liquid recovered from zeolite filtration step (Na_2_O—40 g/L; Al_2_O_3_—3 g/L; ρ—1.04 g/mL). The synthesis, which is carried out for 4 h at 78–88 °C, is followed by a three-step filtration stage and drying at 700 °C. According to the norms of zeolite 4A production, per 1 t of zeolite 1.3000 t of water glass and 1.9660 m^3^ of aluminate liquid are required. Due to a high CEC (5.48 meq/g), this zeolite type is intended for use as a water softener in environmentally friendly detergents^37^. The key function of Alumina plant zeolite is extracting Ca and Mg ions from water, however, it has not yet been tested in terms of Sr sorption capability.

The present study aimed to determine the effect of various process variables on Sr removal (i.e., the contact time, the concentration of Sr, the solution pH, and the presence of competitive species). The applicability of the material for cleaning up contaminated saline environments was examined by varying the percentage content of SW in the liquid phase and by varying the dose of zeolite 4A. Finally, desorption of ^89^Sr sorbed by zeolite 4A from SW was evaluated in deionized water and groundwater, to evaluate the stability of retained radionuclides.

## Results and discussion

### Characteristics of Z4A

**Figure 1:**
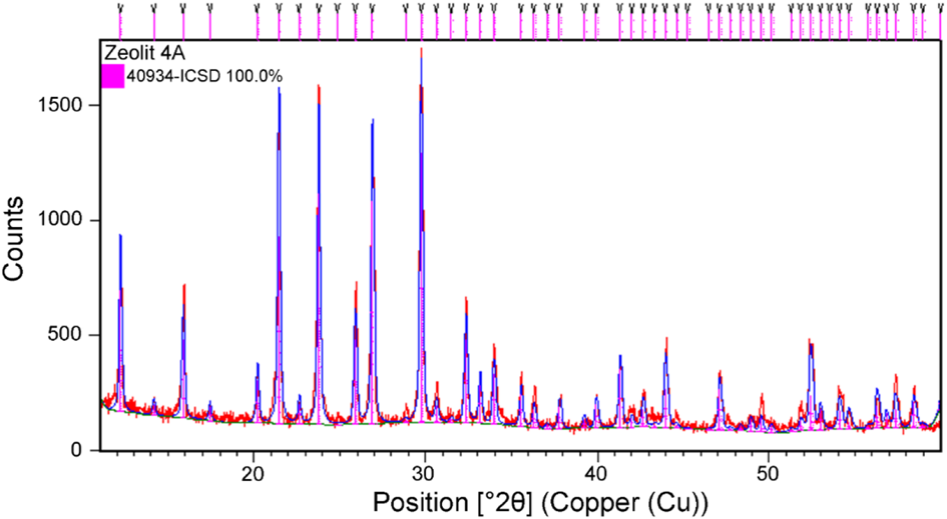
Rietveld structural refinement on synthetic zeolite Z4A. Experimental data are shown in red, while the calculated pattern is given in blue. Magenta vertical lines denote Bragg reflection positions according to the 40934-ICSD standard for 4A zeolite structure.

The chemical and physical properties of the Z4A, specified by the producer, are given in Supplementary material (Table S1). The XRD-based qualitative and quantitative analysis of Z4A crystalline phases has been performed by Rietveld structural refinement (Fig. 1).

As a starting structural model, the zeolite 4A structure has been used (deposited under the number 40934-ICSD^38^). In the course of Rietveld refinement following parameters have been refined: scale factor, unit cell parameters, width profile parameters (U, W, and V) as well as asymmetry parameters. Atomic coordinates and occupancy factors have been constrained as well as thermal factors. The final refinement converged with *R*_wp_ = 8.01%. It was found that Z4A crystallized in cubic space group *Pm*-3*m* with unit-cell parameter *a* = 12.28(1) Å. No additional diffraction peaks were present in the XRD pattern, which pointed out to the sample being single-phase.

**Figure 2:**
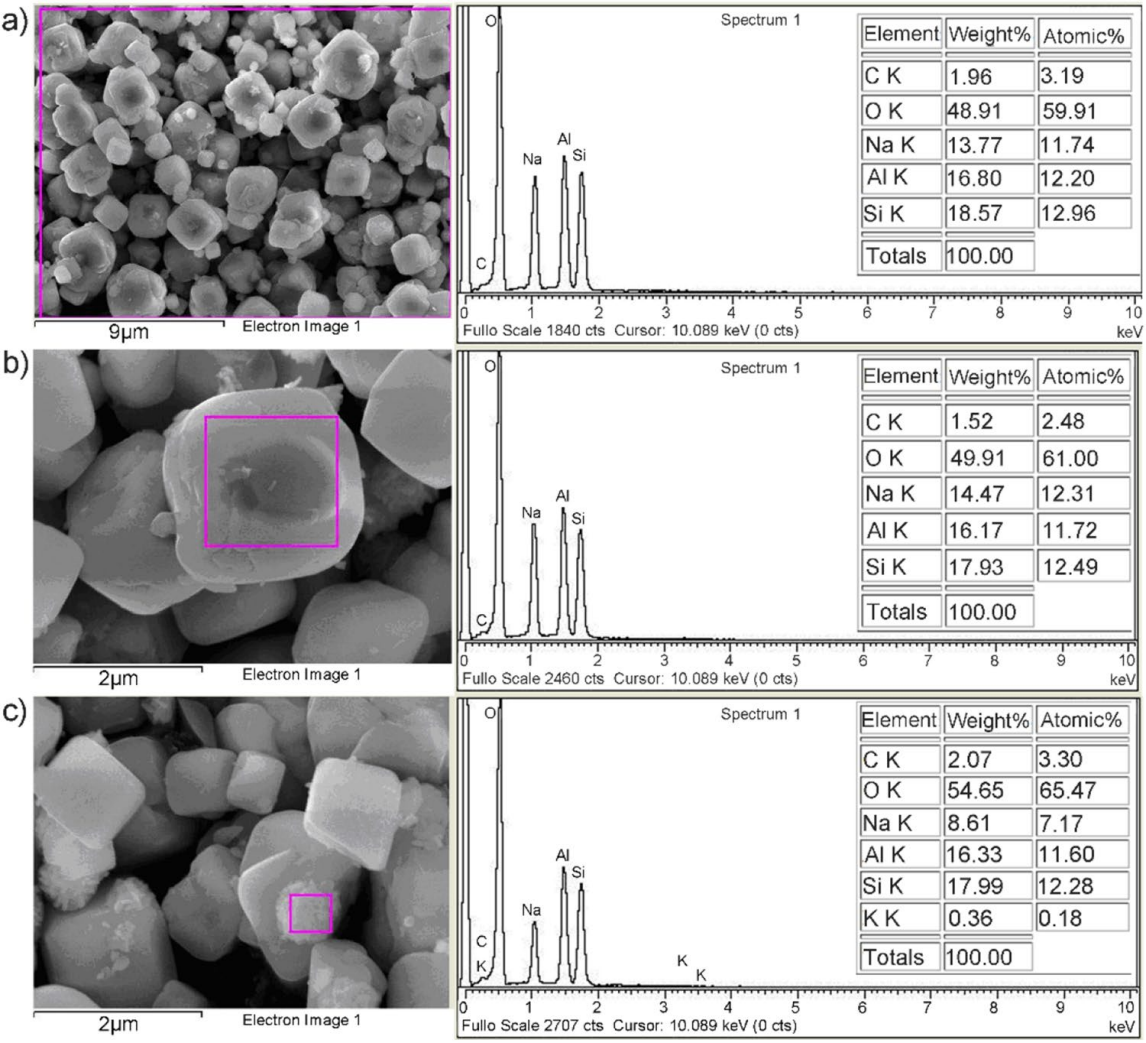
SEM/EDS analysis of Z4A: (**a**) bulk sample, (**b**) isolated cubic particle, and (**c**) isolated spherical particle.

Scanning electronic micrograph of the investigated sample (Fig. 2a) shows the cubic particles typical for zeolite A. The crystals were characterized by truncated edges and apexes (Fig. 2b).

Different morphological entities of zeolite A are the result of the conditions during the synthesis. The formation of cubic crystals with truncated edges and apexes was associated with the excess of aluminum, causing distortion of terminal SiO_4_ tetrahedrons positioned at crystal edges and apexes and the decrease in crystal growth on the $\langle {0\;1\;1} \rangle$ direction^39^. In addition, the spherical aggregates of nano-crystals were observed at higher magnification (Fig. 2c), forming most probably as a result of the spontaneous transformation of zeolite 4A into hydroxy sodalite particles at higher alkalinities^40^. Nevertheless, the characteristic diffraction peaks of the sodalite phase could not be detected in the XDR spectra, which is the evidence of its minor content.

Peaks of the constituent elements (O, Na, Al, and Si) are identified in the EDS spectrum of the Z4A bulk sample (Fig. 2a). The atomic Si/Al ratio calculated from the laterally averaged signal was 1.06, confirming a high degree of isomorphous replacement of Si^4+^ by Al^3+^ ions in the crystalline framework. The exchangeable cation detected in the structure of Z4A was Na (11.74%).

The isolated cubic and spherical particles had the equivalent Si/Al ratio (1.06), however, the atomic ratio of Na in the spherical particles was considerably smaller (7.17%), and the traces of K ions were also detected (0.18%). Based on EDS-spectra analysis, the impurities from the alumina production process could not be identified in Z4A.

### The kinetics of Sr separation

**Figure 3:**
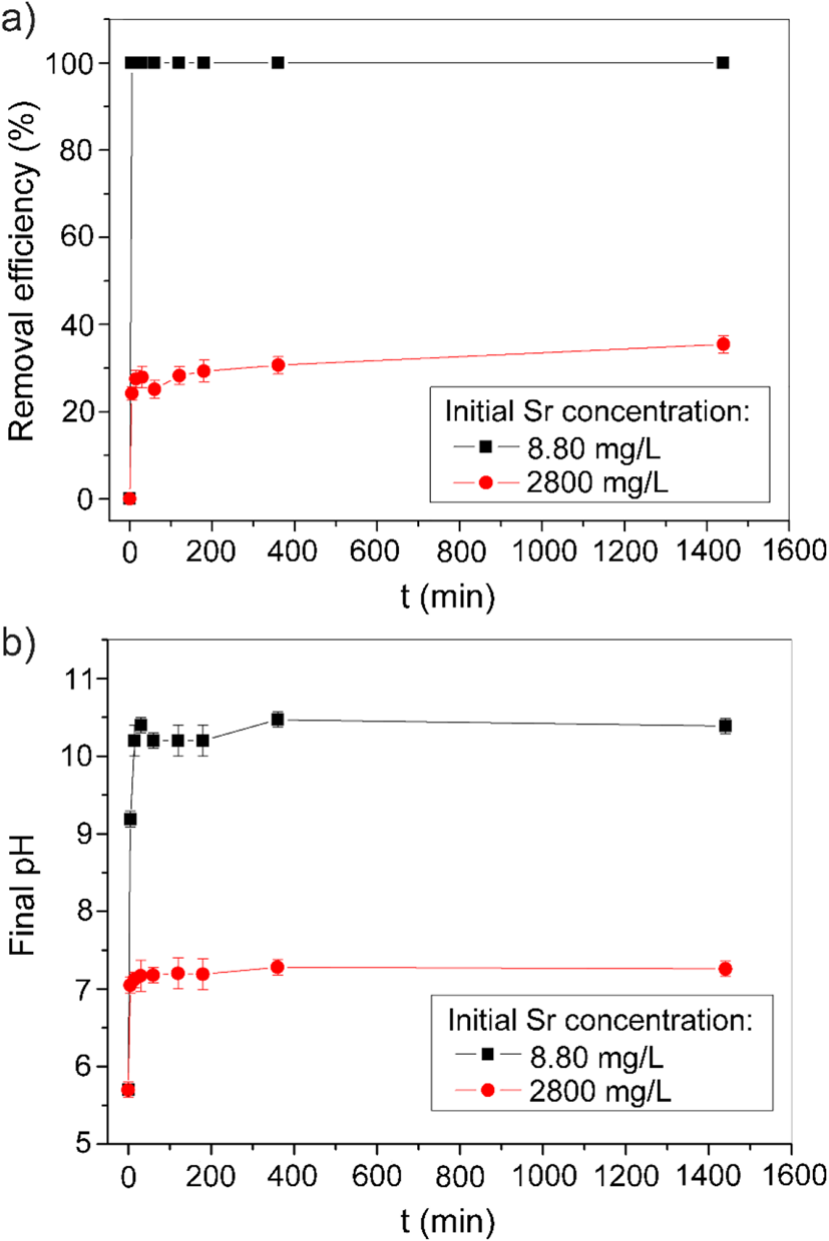
Kinetics of Sr sorption by Z4A from single-component solutions with different initial concentrations: (**a**) process efficiency, (**b**) final pH values.

The efficiency of Sr removal by Z4A against the time is presented in Fig. 3a. After 5 min of contact, Sr was completely removed (100%; 1.76 mg/g) from the solution with initial concentration of 8.80 mg/L, which is typical for SW. The fast and complete removal of Sr from solution with low initial concentration (1 mg/L) was also reported for a commercial synthetic zeolite 4A^31^. In the solution with 2800 mg/L of Sr, the uptake was most rapid in the first few minutes of contact when the gradient between Sr concentration in aqueous solution and Sr concentration at the Z4A surface was the highest. Afterward, the increase in sorbed amounts slowed down, and the equilibrium removal (35%; 200.3 mg/g) was reached within 24 h.

Given that all kinetic data for lower Sr concentration practically corresponded to equilibrium, and the Sr concentrations in the liquid phase were not measurable, it was unfeasible to apply the mathematical models to describe the process's kinetics in more detail. The fast sorption can be attributed to both the low concentration of Sr and the abundance of available active sites on the external surface of Z4A, indicating that the sorption rate is controlled by external mass transfer.

The results obtained for the higher concentration were fitted by models frequently used in the analysis of sorption kinetics: pseudo-first order^41^, pseudo-second order^42^ kinetic model, and the intraparticle diffusion model^43^. The description of the models and the relevant results are given in Supplementary Fig. S1 and Supplementary Table S2. The better agreement of experimental data with the pseudo-second order model than with the pseudo-first order model was obtained (*R*^2^ = 0.998 and *R*^2^ = 0.739, respectively). The amount of Sr sorbed at equilibrium (*q*_e_) calculated using the pseudo-second order equation corresponded to the experimentally obtained value (202.0 mg/g *vs.* 200.3 mg/g), whereas the pseudo-second rate constant (*k*_p2_) and initial sorption rate (*h*) were 2.17 10^–4^ g/mg min and 8.85 mg/g min, respectively. The analysis of the Sr sorption kinetics onto zeolite 4A synthesized from a mixture consisting of aqueous solutions of NaAlO_2_, Na_2_O·SiO_2_, and NaOH^30^, zeolite Na A–X blends synthesized from fly-ash^44^, LTA powder, and LTA-monolith^16^ as well revealed a more accurate description of the ion exchange reaction with the pseudo-second order model. The interparticle diffusion plot exhibited two linear segments with different slopes, i.e., a rapid sorption step (< 30 min), followed by a relatively slow sorption step (30 min–24 h). (Fig. S1, c). The intraparticle diffusion step corresponds to the second linear segment (*R*^2^ = 0.875), from which the intra-particle diffusion rate constant *K*_*id*_ was found to be 1.465 mg/g min^1/2^. A high Sr concentration acts as a driving force in Sr diffusion, and it is evident that intraparticle diffusion increases with the increase in the initial Sr concentration. The linear segment does not pass through the origin, indicating that the boundary layer effects contribute to the process.

The pH of the solution increased instantly after the contact with Z4A (Fig. 3b). At the equilibrium, pH reached the values of 10.4 and 7.2 for lower and higher Sr concentration, respectively. The zeolites of type A are synthesized in an aqueous environment in the presence of NaOH, thus normally exhibit alkaline reaction in water^45^. The influence of ion hydrolysis on the Sr removal can be neglected regardless of the pH increase since the monovalent SrOH^+^ species' contribution becomes significant in solution with pH > 12^46^. However, the results demonstrate that Sr removal from the solution was accompanied by the simultaneous decrease in pH, which is discussed later in the text.

To assure the equilibrium conditions in solutions with variable concentration of Sr, the contact time was set at 24 h in further experiments.

### The effect of initial Sr concentration

**Figure 4:**
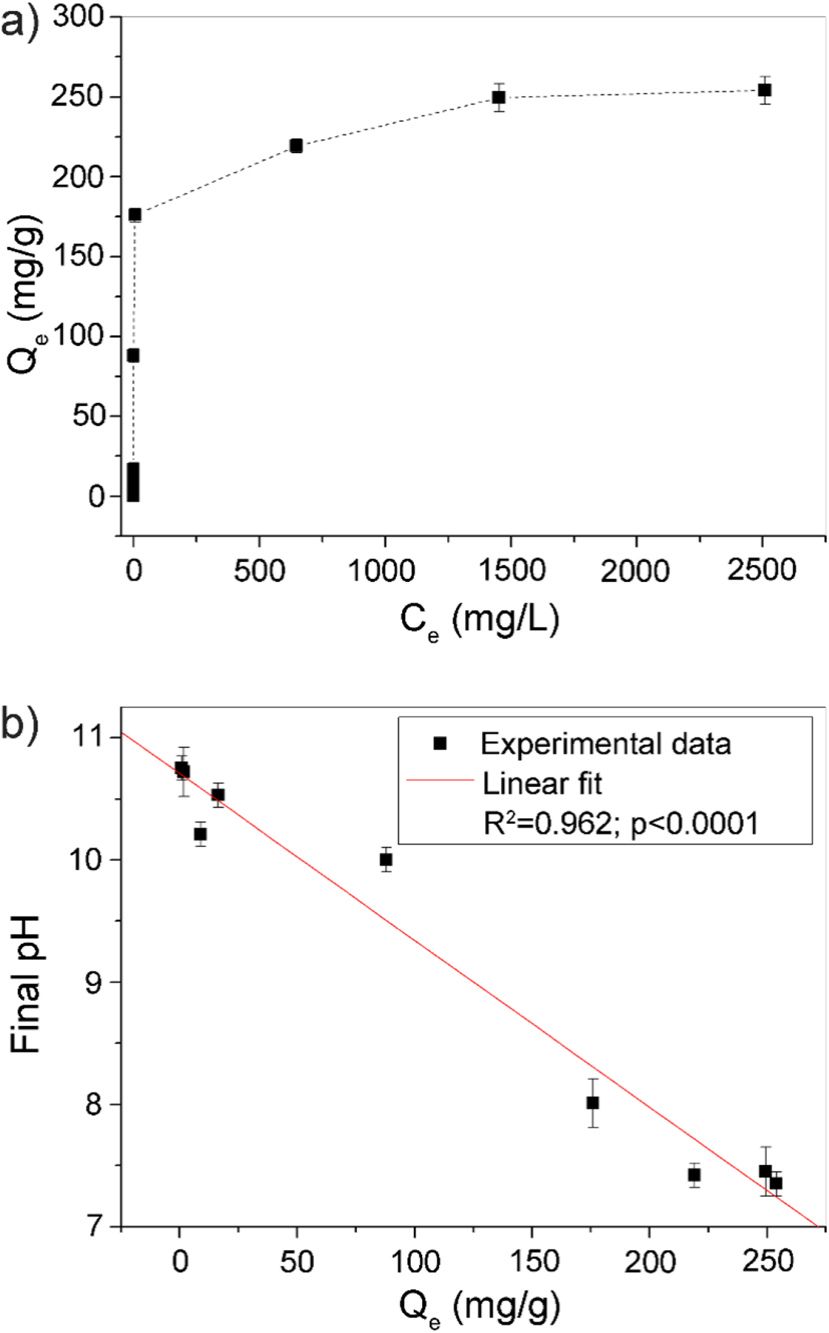
The isotherm of Sr sorption by Z4A (**a**) and the relationship between final pH values and the amounts of Sr sorbed (**b**).

The amounts of Sr, removed by Z4A from the solutions with different initial concentrations, are presented in the form of the sorption isotherm (Fig. 4a). The concentrations of Sr in the liquid and the solid phase at equilibrium conditions are denoted *C*_e_ (mg/L) and *Q*_e_ (mg/g).

The isotherm exhibited the initial increase, whereas the *Q*_e_ values leveled off at higher equilibrium concentrations (*C*_e_). At very low equilibrium concentrations, the sorption isotherms commonly approach a linear form, and the slope of the line is in the positive correlation with the solute affinity towards the solid surface^6^. According to the isotherm classification system based on their shapes^47^, the isotherm of Sr sorption by Z4A can be classified as H2 type (i.e., high affinity class of isotherms with distinctive saturation plateau). The vertical linear portion of the H-type isotherm indicates such high-affinity sorption that in dilute solutions, no measurable amount of the sorbate remains in solution. This suggests that the affinity of Z4A for Sr is remarkable, given that measured *C*_e_ values were below the detection limit (< 0.01 mg/L) for the initial Sr concentrations of 4.40, 8.80 and 44.0 mg/L, 0.10 mg/L for initial Sr concentration of 82.3 mg/L and remained at a very low level (0.15 mg/L) at initiation concentration as high as 440 mg/L.

The data were fitted using linear mathematical models of Langmuir^48^ and Freundlich^49^ (Eqs. (1) and (2), respectively):

1 $$\frac{{C}_{e}}{{Q}_{e}}=\frac{{C}_{e}}{{q}_{max}}+\frac{1}{{q}_{max}{K}_{L}}$$

2 $${\text{ln}}{Q}_{\text{e}}={\text{ln}}{K}_{\text{F}}+1/n\times {\text{ln}}{C}_{\text{e}}$$

The *q*_max_ (mg/g) represents the maximum sorption capacity of zeolites, *K*_L_ (L/mg) is a Langmuir constant which signifies the sorption affinity, while *K*_F_ (mg^1–1/n^L^1/n^g^−1^) and 1/*n* are empirical Freundlich constants associated with the capacity and the affinity of the sorption process. The graphical representation of the fitting is given in the Supplementary Fig. S2, while the calculated parameters are summarized in Table 1.

**Table 1:** The parameters of Sr sorption by Z4A calculated using Langmuir and Freundlich isotherm models.

Langmuir model		Freundlich model	
*q*_max_ (mg/g)	252.5	1/*n*	0.191
*K*_L_ (L/mg)	0.084	*K*_F_ (mg^1–1/n^L^1/n^ g^−1^)	66.80
*R*^2^	0.998	*R*^2^	0.692

Removal of Sr was better described using the Langmuir (*R*^2^ = 0.998) than the Freundlich (*R*^2^ = 0.692) model, and the maximum sorption capacity predicted by the Langmuir equation was equivalent to the value obtained experimentally. The agreement with the Langmuir model equation implies that the active centers on the surface of Z4A are fairly homogeneous for Sr and that monolayer sorption takes place, resulting in the clearly defined maximum capacity.

Considering Sr removal from single-component solutions, the *q*_max_ of the sample Z4A was higher compared to the capacity (*q*_max_ = 204 mg/g) of the commercial zeolite 4A, Sigma-Aldrich^29^, and lower compared to the capacity (*q*_max_ = 303 mg/g) of the zeolite A synthesized in the laboratory^30^. The observed inconsistency in *q*_max_ between three zeolite 4A samples reflects the influence of sources and conditions during synthesis onto chemical, structural, surface and sorption properties of zeolite, and justifies the need for characterization of products obtained by different preparation routes. Nevertheless, the zeolite obtained from the Bayer process solutions can compete with other synthetic zeolites in terms of Sr sorption capacity. It is important to emphasize that the capacity of Z4A far exceeds capacity of many novel materials intended for Sr removal: biogenic apatite (47 mg/g^17^), TiCaMg phosphate (172 mg/g^50^), zero valent iron nanoparticles–zeolite (nZVI–Z) and nano-Fe/Cu–zeolite (nFe/Cu–Z) (84.12 mg/g and 88.74 mg/g^23^), MnO_2_-alginate beads (102 mg/g^21^), and barium-sulfate-impregnated reduced graphene oxide aerogel (232.89 mg/g^51^).

The pH values measured in the supernatants after the sorption experiments (Fig. 4b) fluctuate with the increase in *Q*_e_, from pH 10.7 to pH 7.2. In addition to being negative, the dependence between the final pH and *Q*_e_ was liner (*R*^2^ = 0.962).

Zeolites with a lower Si/Al ratio exhibit higher selectivity for H^+^ due to a higher negative charge density^52^. It was found that zeolite 4A binds considerable amounts of H^+^ even at neutral and weakly alkaline pH region. H^+^ is bound through hydrogen bonding, which becomes very strong as it occurs at the negatively charged sites in the zeolite 4A. Due to Lewis acid–base reactions, hydrogen bonding with bridging oxygen of the Al–O–Si linkage in the zeolite structure results in a covalent bonding and formation of Al–OH–Si sites^52^. The increase in H^+^ concentrations in the surrounding medium after Sr removal further confirms the participation of functional groups on the surface of Z4A capable of forming complexes with Sr according to the reaction:

$$= {\text{O}}{-}{\text{H }} + {\text{Sr}}^{{{2} + }} \to \, = {\text{O}}{-} {\text{Sr}}^{ + } + {\text{ H}}^{ + }$$

The specific sorption of cations, followed by a decrease in solution pH, was identified as an operating cation removal mechanism by natural zeolites^53,54^. Furthermore, the decrease in the O–H, Si–O, and Al–O bands intensities and the shifts in their positions were detected in the Fourier transform infrared (FTIR) spectra of zeolite 4A exchanged with Cu ions, and these changes were attributed to the strong interaction of Cu ions with the related surface groups^55^.

**Figure 5:**
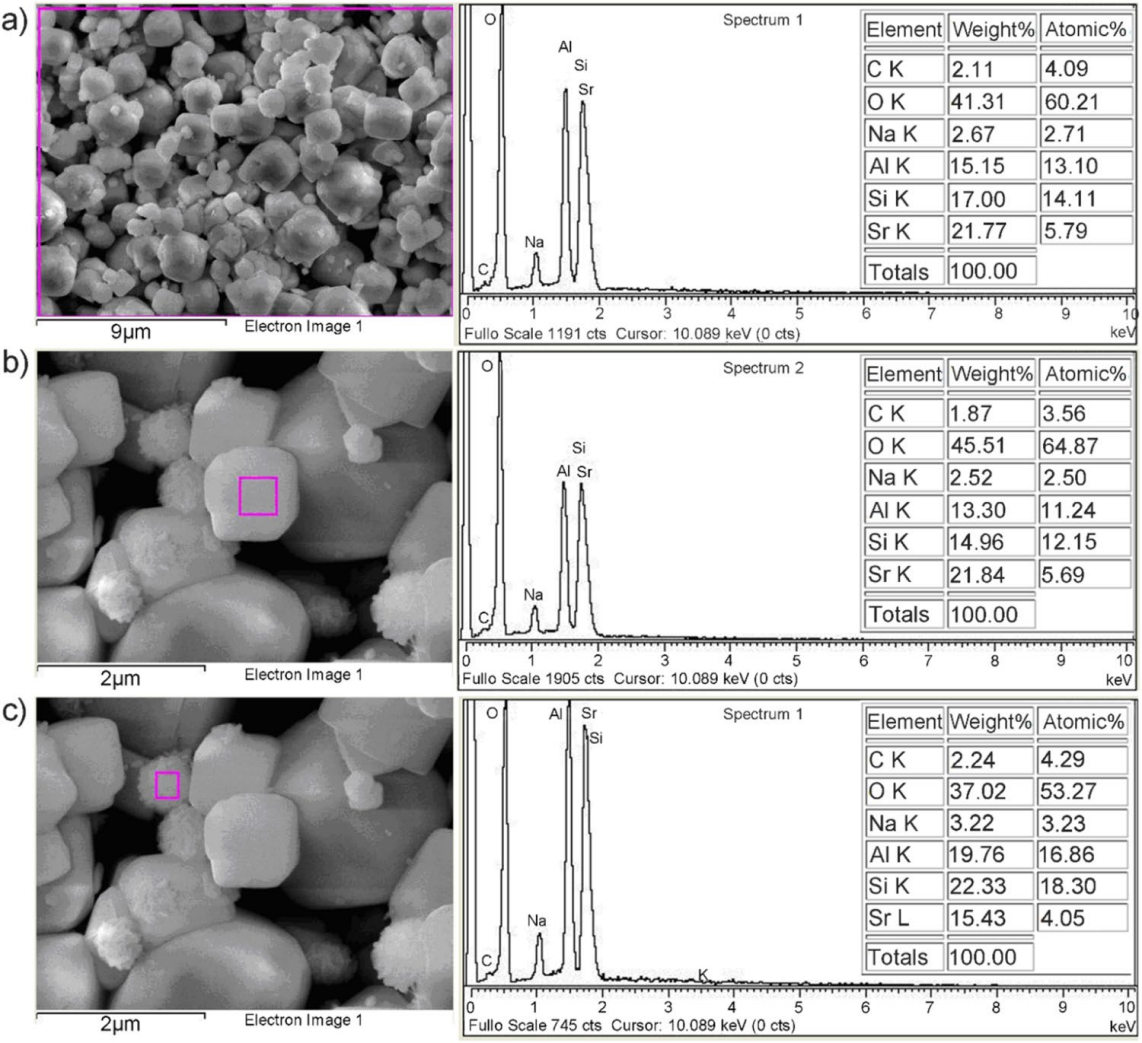
SEM/EDS analysis of Z4A after saturation with Sr: (**a**) bulk sample, (**b**) isolated cubic particle, and (**c**) isolated spherical particle.

The morphology and the composition of the Z4A particles saturated with Sr are presented in Fig. 5. The morphology of Z4A particles (Fig. 2a) was preserved after Z4A saturation with Sr (Fig. 5a). For comparison, the cubic particles of zeolite 4A were transformed into irregular and spherical-like particles with rough surfaces after the sorption of Cu ions^55^, which implies that Z4A structure provides better accommodation for Sr.

A significant decrease in the atomic ratio of Na in Z4A (from 11.74 to 2.71%) and a simultaneous rise in Sr atomic ratio to 5.79%, were determined by EDS analysis (Fig. 5a). The comparison of the EDS-spectra presented in Fig. 5b,c has disclosed a lower affinity of spherical aggregates (hydroxy sodalite particles) towards Sr (4.05%). The results demonstrate that the exchange with Na ions was a main operating mechanism in Sr removal, according to the reaction:

$${\text{Sr}}^{{{2} + }}_{{({\text{s}})}} + {\text{ 2Na}}^{ + }_{{({\text{Z4A}})}} \leftrightarrow {\text{ Sr}}^{{{2} + }}_{{({\text{Z4A}})}} + {\text{ 2Na}}^{ + }_{{({\text{s}})}}$$

where s and Z4A denote solution and zeolite 4A phase, respectively.

### The effect of initial pH

**Figure 6:**
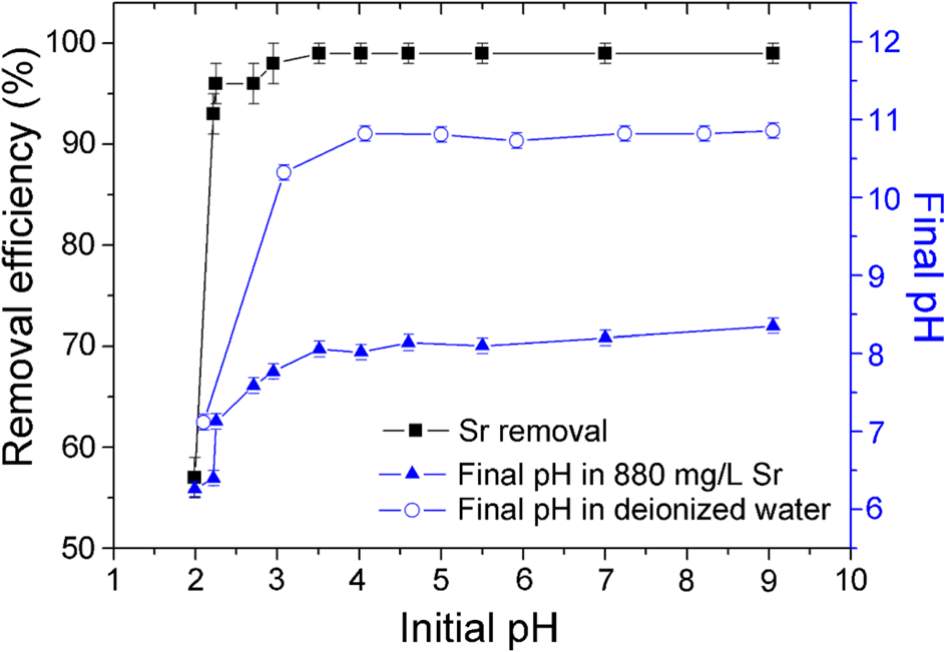
The effect of initial pH on the Sr removal efficiency by Z4A, the final pH values in Sr-containing solutions and the final pH values in the blank solutions.

The influence of initial solution pH on Sr removal by Z4A is given in Fig. 6. The substantial increase in Sr sorption from 53 to 99% (93.3–174.2 mg/g) was observed over a narrow range of initial pH values (from pH 2.0 to pH 3.5). With the further rise in initial pH (4.0–9.0), Sr removal remained at a constant level.

In acidic environments, H^+^ ions compete with metal cations for sorption on zeolites via ion exchange of extra framework cations^56^. Since the effect of competition depends on the concentration of ions in the solution, the removal of Sr is lower if the H^+^ ion concentration is high. Moreover, acidic pH values may affect cation sorption adversely by provoking structural changes of the zeolite. The structure of zeolite 4A is unstable at pH < 4.0^37^, while the rapid and stoichiometric dissolution of zeolite 4A followed by the precipitation of silicate occurs at pH < 1.5^57^. Therefore, both the competition with H^+^ ions and the instability of the Z4A crystal framework contribute to the observed decrease in Sr sorption with the initial pH decrease below pH 4 (Fig. 6).

The final (equilibrium) pH values in the systems without and with Sr are compared in Fig. 6, for different initial pH values. The consumption of protons from the blank solutions by Z4A gave a substantial rise in the final pH with respect to initial pH. As the initial pH increased from pH 2.0 to pH 4.0, the final values increased from pH 7.1 to pH 10.8. With the further increase in initial pH, the final pH values were buffered after reaction with Z4A to the constant value of 10.8.

The pattern of final pH values measured in Sr-containing solutions was similar to that obtained in deionized water, however, the absolute pH values were lower (from pH 6.2 to pH 8.2). The changes observed in respect to the initial pH values are the result of complex interactions of the Z4A surface with both the aqueous medium (consumption of H^+^) and the Sr (release of H^+^). The increase in Sr sorption was related to the increase in equilibrium pH, and the plateau of Sr removal coincides with the plateau of final pH values (pH 8.1 ± 0.1), obtained for a range of initial pH (pH 3.5–pH 9.0). The ability of different synthetic zeolite samples to buffer the initial solution pH (pH 4–pH 10) to near neutral final pH was observed in the study of Ca removal and attributed to the presence of acidic and basic sites on their surface^58^. Our results further signify that the buffering capacity of Z4A makes the Sr separation independent of the initial pH values in a broad range, which provides practical benefits over the sorbents with efficiency significantly related to this variable^24^. Comparably, the positive correlation between Sr sorption yield and equilibrium pH was detected in the work of Lee et al.^15^, where the best performance of the zeolite 4A was achieved at equilibrium pH ~ 8.0. The overall results indicate that the initial pH of the contaminated solution should be pH ≥ 4 for the best performance of Z4A.

### Competitive sorption

**Figure 7:**
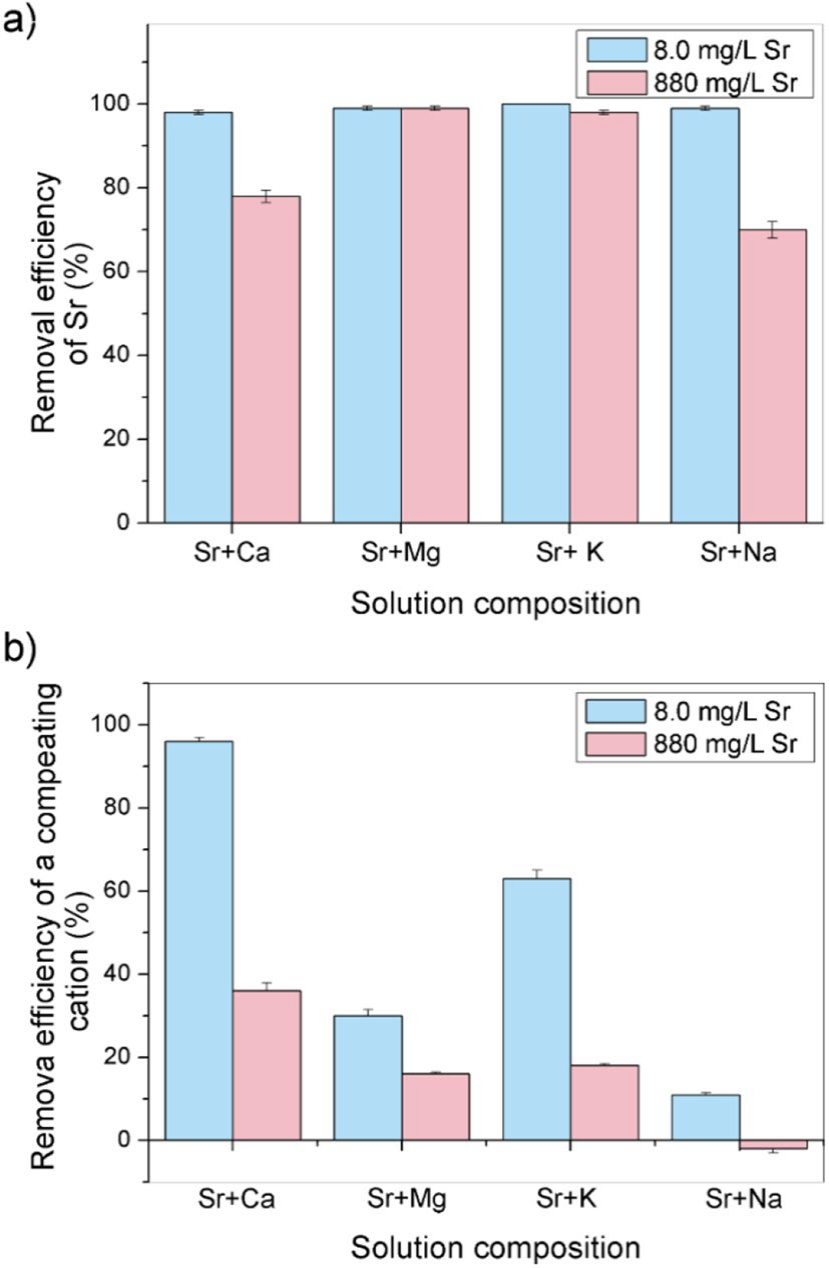
Removal efficiency of Sr (**a**) and the competing cation (**b**) from bi-component solutions having initial Sr concentrations of either 8.0 mg/L or 880 mg/L and competing cation concentration typical for SW (420 mg/L Ca, 1200 mg/L Mg, 320 mg/L K, and 10,600 mg/L Na).

The results obtained by determining the Sr sorption efficiency in bi-component solutions with interfering cations at their characteristic concentrations in SW are shown in Fig. 7a. The concentration of Sr was either 8.0 mg/L or 880 mg/L. Furthermore, the sorption efficiency of each interfering cation was determined, and the results are shown in Fig. 7b.

The ions of Sr were almost completely removed by Z4A from bi-component solutions when the mass ratio of the two cations was typical for SW. The removal efficiency was 98% (1.57 mg/g) in the presence of Ca, 99% (1.58 mg/g) in solutions with Mg and Na, and 100% in K-containing mixture (1.60 mg/g). In parallel, 96% Ca (80.6 mg/g), 75% K (48.0 mg/g), and 32% Mg (76.8 mg/g) were bound to Z4A. Giving its highest initial concentration with respect to other cations in SW, and the amount already incorporated in the structure of Z4A, overall Na content was reduced by 10% (212 mg/g). The experiments show that the effect of individual competing cations from SW on the Z4A capacity to retain Sr was negligible, although Sr concentration in all investigated systems was far smaller than the concentration of a competitive ion. Given that a considerable amount of each competitive ion remained in solution, the large capacity of the Z4A is not the argument for explaining such a small effect of the competition, but rather the selectivity of Z4A structure towards Sr.

The presence of Mg and K virtually did not affect Sr binding by Z4A (99% and 98%; 174.2 mg/g and 172.5 mg/g, respectively) even if the Sr concentration was initially high (880 mg/L). Moreover, the higher concentration of Sr suppressed the removal of Mg and K to 16% (38.4 mg/g) and 18% (11.5 mg/g), respectively. The removal efficiency of Sr decreased to 78% (137.3 mg/g) and 70% (123.2 mg/g) in combination with Ca and Na ions, respectively. Simultaneously, 38% of Ca (31.9 mg/g) was sorbed from the binary mixture, while the final concentration of Na in the solution increased (i.e., more Na was exchanged with Sr than retained in the Z4A structure).

**Figure 8:**
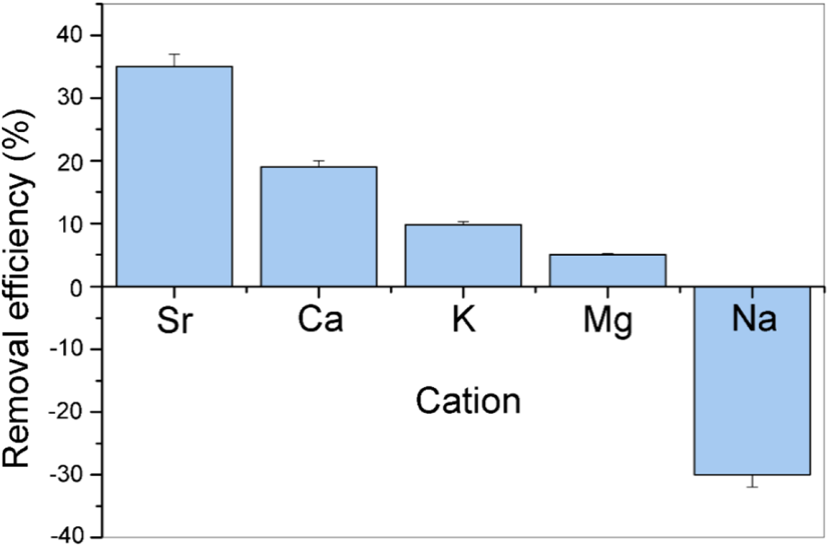
Competitive removal of Sr, Ca, K, Mg, and Na from the equimolar five-component mixture (0.02 mol/L of each cation) by Z4A.

The selectivity of Z4A towards major cation in SW was further investigated in a more complex five-component solution having equimolar (0.02 mol/L) concentrations of Na, Mg, Sr, Ca, and K (Fig. 8).

The selectivity of Z4A decreased in order: Sr > Ca > K > Mg > Na (i.e., the sorption capacities decreased in the order 1.398 mmolSr/g, 0.758 mmolCa/g, 0.392 mmolK/g, and 0.200 mmolMg/g). This trend was confirmed by the analysis of the EDS spectrum of the Z4A sample after interaction with the mixture solution (Supplementary Fig. S3), showing the decrease in atomic percentages on the Z4A surface in the order Sr (3.72%) > Ca (1.71%) > K (0.95%) > Mg (not detectable due to its low surface concentration). Although divalent Sr and Ca have similar ionic radii and chemical properties, obtained results strongly indicate that Z4A is more selective toward Sr. In the investigated system where the molar concentrations of all cations were equal, Sr sorption from the solution was 1.8 times higher than Ca, while 3.6 and 7.0 times higher compared to K and Mg. Comparable element ratios were obtained by Z4A surface analysis (Sr/Ca = 2.1, Sr/K = 4.0, Supplementary Fig. S3). Giving that the primary mechanism of Sr, Ca, Mg, and K removal from the mixture is the exchange with Na ions, aqueous Na concentration increased (Fig. 8), while Na concentration in the soil phase decreased accordingly (Supplementary Fig. S3). The selectivity of Z4A towards investigated cations is in agreement with the sequence (Sr > Ca > Mg > Na) deduced from the equilibrium isotherm data^59^.

### The influence of water salinity

**Figure 9:**
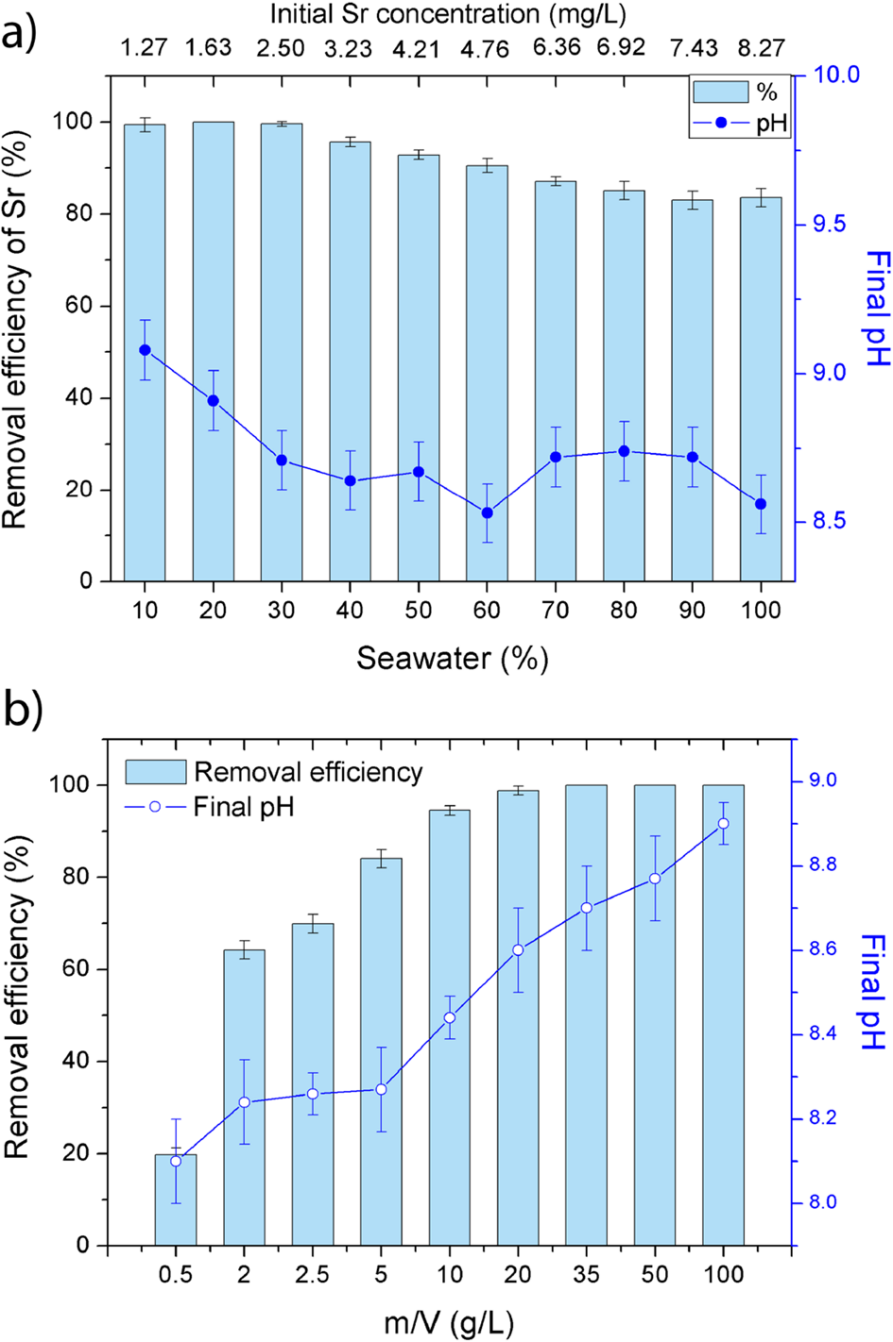
(**a**) The effect of seawater concentrations on the efficiency of Sr separation by Z4A (**a**), and (**b**) the effect of the solid-to-solution ratio on the efficiency of Sr removal from SW and final pH values.

The composition of model seawater (SW) is given in Supplementary Table S3. The influence of water salinity on the efficiency of Sr separation is presented in Fig. 9a. Concerning the initial Sr concentration, the sorption was complete when the ratio of SW in the sample was ≤ 30% (0.25 mg/g at 10% SW, 0.33 mg/g at 20% SW, and 0.50 mg/g at 30% SW), whereas it gradually shifted from 96% (0.62 mg/g) to 84% (1.39 mg/g) with the increase of SW content from 40 to 100%. Sorption efficiency of Sr was evidently influenced by co-existing cations, which are present in SW in much higher concentrations.

The removal of Sr from SW (84%) was lower compared to 100% removal under the same set of experimental conditions using a single-component Sr solution and compared to the 98–100% efficiency in bi-component solutions with the major coexisting cations. The complexity of the SW matrix led to the reduced Sr uptake by Z4A through the synergic effect of all cationic species which compete for the sorption sites. The analysis of the SW after equilibration with Z4A showed the removal of 45% of Ca (39.6 mg/g), 14% of K (11.2 mg/g), and 12% (31.4 mg/g) of Mg relative to their initial concentrations (Supplementary Table S3), demonstrating the ability of Z4A for hardness removal in seawater. Comparable results regarding Ca and Mg removal from seawater (around 45% and 10%, respectively) were reported for zeolite A samples synthesized from the waste aluminum and disposed of silica gel^58^.

Even though the efficiency of Sr sorption decreased with the increasing complexity of the solution, the Z4A affinity for Sr was still very high and particularly stands out when compared to the natural zeolite for which complete blockage of Sr removal was detected at 70% SW and above^17^. The removal of Sr reached maximally 62.3% in seawater media using 5 g/L of composite magnetic nanoparticles (CuFe_2_O_4_)^24^, 40.1% using zeolite-alginate foam^22^, and 44.3% using MnO_2_-alginate beads^21^.

The efficiency of Sr removal from SW obtained at different Z4A doses is presented in Fig. 9b. With the increase in Z4A dose from 0.5 to 20 g/L, the removal efficiency was improved and increased from 20 to 99%, whereas the sorbed amount of Sr per gram of Z4A decreased from 3.30 to 0.41 mg/g. The complete separation (100%) achieved with the higher investigated Z4A doses was followed by the decrease in Sr amounts per unit mass of Z4A (i.e., 0.24 mg/g, 0.16 mg/g, and 0.08 mg/g, for the doses of 35 g/L, 50 g/L, and 100 g/L, respectively). In the literature, more than 99% removal of Sr at the initial concentration in SW of 0.2 mg/L was reported using > 20 g/L of zeolite 4A and 5 g/L of Ba-impregnated zeolite 4A^15^. Given that the target efficiency for decontamination is usually 99% of the ^90^Sr in solution (i.e., targeted decontamination factor (DE) is 100)^14^, the results from Fig. 9a,b, demonstrate that the dose of Z4A can be successfully optimized for water salinity so that Sr activity is reduced to the required level.

**Figure 10:**
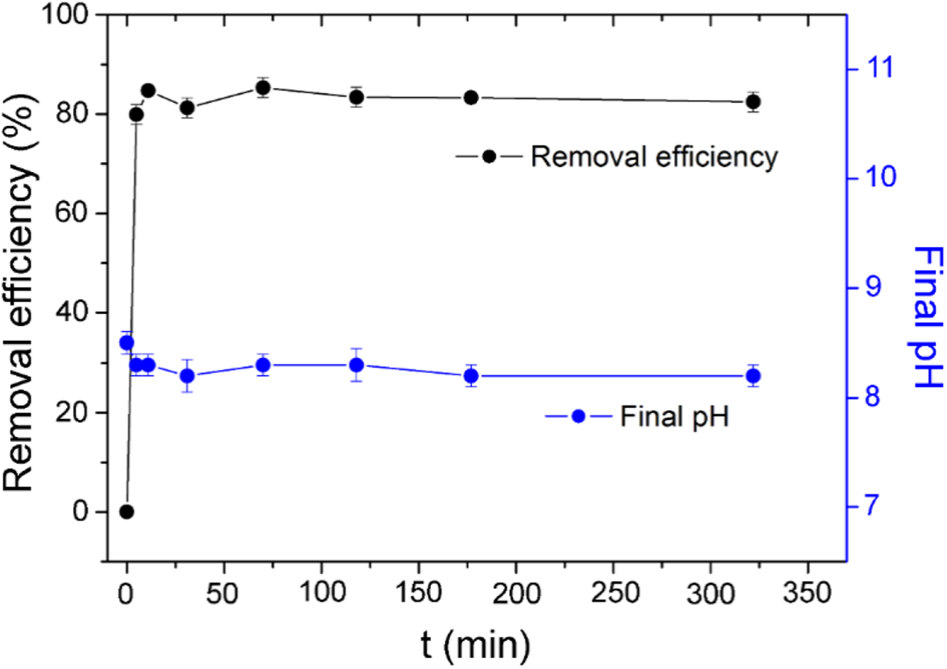
The effects of contact time on the efficiency of Sr removal from SW and final pH values.

The kinetic study shows that, upon Z4A addition, Sr removal in SW takes place practically at the same rate as in a single-component solution (Fig. 10), i.e., the equilibrium was accomplished within the first 5–10 min of contact. The pH of SW exhibited a slight decrease upon reaction with Z4A, from initial pH 8.4 to equilibrium pH 8.2.

### Desorption of Sr ions

Adsorption yield of Sr from SW solution measured by radioisotope tracer (^89^Sr) was 84% (1.39 mg/g), indicating equivalent sorption affinity of Z4A towards the stable and radioactive Sr isotopes.

The desorbed fractions of Sr were extremely low using both deionized water and groundwater (Supplementary Table S3), at the same solid-to-solution ratio (5 g/L) (Table 2). In deionized water, only 0.1–0.2% of initially sorbed ^89^Sr was released during the period of 60 days. Desorption equilibrium was reached after approximately nine days of contact with groundwater. Despite the high concentration of cations, especially Ca (Supplementary Table S3), maximally 0.7% (0.01 mg/g) of Sr was desorbed.

**Table 2:** Time-dependent desorption of ^89^Sr from Z4A solid residue after reaction with SW.

Leaching time (days)	Desorption efficiency (%)								
	1	4	9	13	22	28	33	45	60
Deionized water	0.1	0.2	0.1	0.1	0.2	0.1	0.1	0.1	0.1
Groundwater	0.1	0.5	0.7	0.7	0.6	0.7	0.7	0.7	0.7

The release of radionuclides from the waste forms may occur during processing, storage, and disposal of nuclear materials. In particular, the stability of radionuclides is important for the safety of long-term disposal of nuclear waste, as the sub-surface facilities may become infiltrated with groundwater^60^. The results obtained from desorption experiments demonstrate that Sr radionuclides are tightly held by Z4A. Furthermore, to ensure a higher degree of radionuclide retention, saturated ion-exchangers are treated as radioactive waste and commonly are incorporated in cement, bitumen, glasses, and ceramic waste forms^5^, as well as in the geopolymer matrix^61^. Being an inorganic, aluminosilicate material, compatible with most of the solidification matrices, the Z4A has good prospects for safe disposal.

## Materials and methods

### Characterization of Z4A

The sample denoted Z4A, supplied from the Zvornik Alumina Refinery, was used without additional treatments or modifications. The crystalline structure of Z4A was determined by X-ray diffraction (XRD) analysis using Philips MPD 1880, equipped with a proportional counter. The data were collected in the 10–60 2Θ range, and the phases were identified using the Inorganic Crystal Structure Database FIZ Karlsruhe (ICSD database). The JEOL thermal field emission scanning electron microscope (FE-SEM, model JSM-7000F), was used to observe the morphology of the particles of neat Z4A, and Z4A after reaction with Sr and more complex five-component solution. The FE-SEM was linked to an Oxford Instruments EDS/INCA 350 energy dispersive X-ray spectra (EDS) analyzer for elemental analysis. The prime EDS spectral lines of Si Kα (1739 keV) and Sr Lα (1806 keV) are closely positioned, which can significantly affect the atomic concentration results of Si and Sr due to the limited spectral resolution of the EDS detector (~ 70 eV at Si Ka). Consequently, the contributions of the two spectral lines overlap, forming a convoluted broader asymmetric spectral feature. Several steps have been taken to minimize the error of the quantitative results in processing the EDS spectra. EDS spectra have been recorded with prolonged time to get a good amount of counts and well-defined spectral lines with 5 eV/channel sampling resolution. The spectra' subsequent deconvolution was carefully performed using data from NIST X-ray Transition Energy Database, according to which the spectrometer has also been calibrated on standard samples.

### Sorption experiments

The study of Sr removal by Z4A was conducted in batch conditions, at ambient temperature (21 ± 1 °C). The model single- and multi-component solutions were prepared by dissolving the nitrate salts of Sr, Ca, Mg, K, and Na (Fisher Scientific, p.a. purity) in deionized water.

When specified, initial pH values were adjusted by the dropwise addition of either NaOH or HNO_3_ solutions. Initial pH values, as well as the final, were measured by InoLab WTW pH meter. Synthetic SW (Supplementary Table S3) was prepared by dissolving 36.0 g of the aquarium salt mix (Coral pro salt mix—Red Sea) in 1 L of deionized water.

Suspensions of Z4A in specified solutions were prepared in 50 mL centrifuge tubes, agitated on bench-top overhead laboratory shaker at 10 rpm for a certain amount of time, and subsequently subjected to the solid–liquid separation using Heraeus Megafuge 16 (Thermo Scientific) at 9000 rpm for 10 min.

The kinetics of the Sr removal was explored using Sr solutions with initial concentrations of 8.80 mg/L and 2800 mg/L. The lower concentration was selected based on Sr concentration in SW, while the rate of sorption at higher concentration was essential for assessment of equilibrium times in the study of other factors. The solid-to-solution ratio was 5 g/L, the initial pH was 5.7 ± 0.1, and the contact time was varied in the range 5 min–24 h.

The effect of initial Sr concentration was studied in the range from 4.40 to 3780 mg/L. Based on the kinetic experiments, the contact time of 24 h was chosen as sufficient for the equilibrium sorption. The solid-to-solution ratio was 5 g/L. The initial pH values of Sr solutions were in the range pH 5.5–pH 5.8, and they were used without further pH adjustments.

Solution pH effect on the efficiency of Sr removal was examined using the fixed Sr concentration of 880 mg/L, solid-to-solution ratio (5 g/L), and equilibration time (24 h), while varying the initial pH in the range 2.0–9.0. The series of blank experiments were performed under the same experimental conditions using deionized water, to determine the effect of Z4A itself on the solution pH.

The effect of coexisting cations in SW (Na, K, Ca and Mg) on Sr removal efficiency by Z4A was firstly investigated in bi-component solutions having initial Sr concentrations of either 8.0 mg/L or 880 mg/L, while the concentration of other cation was analogous to that of SW (Supplementary Table S3). Furthermore, the solution with equimolar initial concentrations of each cation (0.02 mol/L of Na, K, Sr, Ca, and Mg) was applied to disclose Z4A selectivity towards major cationic species in SW. In all experiments, the suspensions (5 g/L) were agitated for 24 h.

The influence of water salinity (0–100% synthetic seawater) on the Sr removal efficiency was explored at the 5 g/L dose of Z4A, following 24 h of contact. The effect of sorbent mass (0.5–100 g/L) on Sr removal was examined using variable amounts of Z4A (0.1–1 g) in the fixed volume of SW (20 mL). The effect of time on Sr removal from SW was studied using the same conditions as for single-component solutions.

The concentrations of metals were measured by Atomic Absorption Spectrometer (AAS) Perkin Elmer 3100. The calibration standards for the determination of Sr concentration in SW were prepared with artificial seawater solution (10,000 mg/L Na, 1250 mg/L Mg, 400 mg/L Ca, and 400 mg/L K)^62^. The experiments were performed in duplicate for each experimental setup, and the results are presented as mean values.

### Desorption experiments

Desorption experiments were performed to investigate the stability of Sr sorbed from SW by Z4A. Firstly, Sr was sorbed on Z4A from artificial SW spiked with ^89^Sr certified standard solution (100 kBq/g, relative uncertainty 0.7%, reference date: 19.8.2019.) provided by Czech Metrology Institute (Prague, CZ), using the solid-to-solution ratio of 5 g/L. After centrifugation, saturated Z4A solids were mixed either with 20 mL of deionized water (0.05 µS/cm) or with groundwater (Supplementary Table S3) from the location Šibice (Zaprešić, Croatia). Aliquots of supernatant were taken periodically for 60 days to follow ^89^Sr desorption from Z4A. The activity of ^89^Sr was measured by Cherenkov counting using liquid scintillation counter Tri-Carb 3180 TR/SL (PerkinElmer, USA).

## Supplementary Information

Supplementary Information.
