# Supplementary material for: Efficient separation of strontium radionuclides from high-salinity wastewater by zeolite 4A synthesized from Bayer process liquids
Source: Sci Rep. 2021 Jan 18;11:1738. doi: 10.1038/s41598-021-81255-y (PMC7814134; doi:10.1038/s41598-021-81255-y)
Supplement: Supplementary file 1 — Supplementary Information. [file 41598_2021_81255_MOESM1_ESM.doc]

**Supplementary information**

**Efficient separation of strontium radionuclides from high-salinity wastewater by zeolite 4A synthesized from Bayer process liquids**

**Ivana Smičiklas^1^, Ivana Coha^2*^, Mihajlo Jović^1^, Marijana Nodilo^2^,**

**Marija Šljivić-Ivanović^1^, Slavko Smiljanić^3^ & Željko Grahek^2^**

*^1^„VINČA” Institute of Nuclear Sciences - National Institute of the Republic of Serbia, University of Belgrade, Mike Petrovića Alasa 12-14, 11001 Belgrade, Serbia*

*^2^Ruđer Bošković Institute, Bijenička cesta 54, 10000 Zagreb, Croatia*

*^3^University of East Sarajevo, Faculty of Technology, Karakaj 34A, 75400 Zvornik, Republic of Srpska, Bosnia and Herzegovina*

^*^ Corresponding author: [ivana.coha@irb.hr](mailto:ivana.coha@irb.hr)

| Properties | Units | Values |
| --- | --- | --- |
| Water content (1 h, 1000^o^C) | % | 19.01 |
| Ion-exchange capacity | mgCa/g | 114.4 |
| Al_2_O_3_ | % | 36 |
| Na_2_O | % | 22 |
| SiO_2_ | % | 42 |
| Iron (total) | ppm | 103 |
| Bulk density | g/L | 302 |
| pH value (10%, 25°) |  | 11.22 |
| Particle size distribution  Mean  Greater than 10 µm | µm  % | 4.8  6.8 |

**Table S1.** Chemical and physical properties of Z4A.

**Modeling the kinetic data**

The kinetic data of Sr sorption by Z4A were fitted by the pseudo-first (equation (S1))^1^ and pseudo-second order (equation (S2))^2^ kinetic models, widely used to analyze the kinetics of the sorption processes:


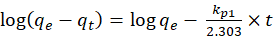
 (S1)


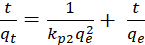
 (S2)

where *q_e_* and *q_t_* (mg/g) denote the amounts (mg/g) of Sr sorbed by Z4A at equilibrium and time *t* (min), while *k_p1_* (1/min) and *k_p2_* (g/mg∙min) denote the pseudo-first order and pseudo-second order rate constants. From equation (S2), when *t*→0, the initial sorption rate *h* (mg/g∙min), can be calculated as follows:

*h* = *k_p2_q_e_^2^* (S3)

Additionally, the data were fitted using the intra-particle diffusion model^3^:

*q_t_* = *K_id_* t^1∕2^ + C (S4)

In the equation (S4), *K_id_* represents the intra-particle diffusion rate constant (mmol/g^.^min^1/2^), whereas C (mg/g) is a parameter proportional to the boundary layer thickness. The intra-particle diffusion is the rate limiting step of the sorption process if the straight-line dependency *q_t_* vs *t^1/2^* passes through the origin. Since several diffusion processes can characterize the transfer of sorbate, the intra-particle diffusion plot may exhibit multilinearity.

The results of the data fitting are presented in Fig. S1, and calculated kinetic parameters are summarized in Table S2.


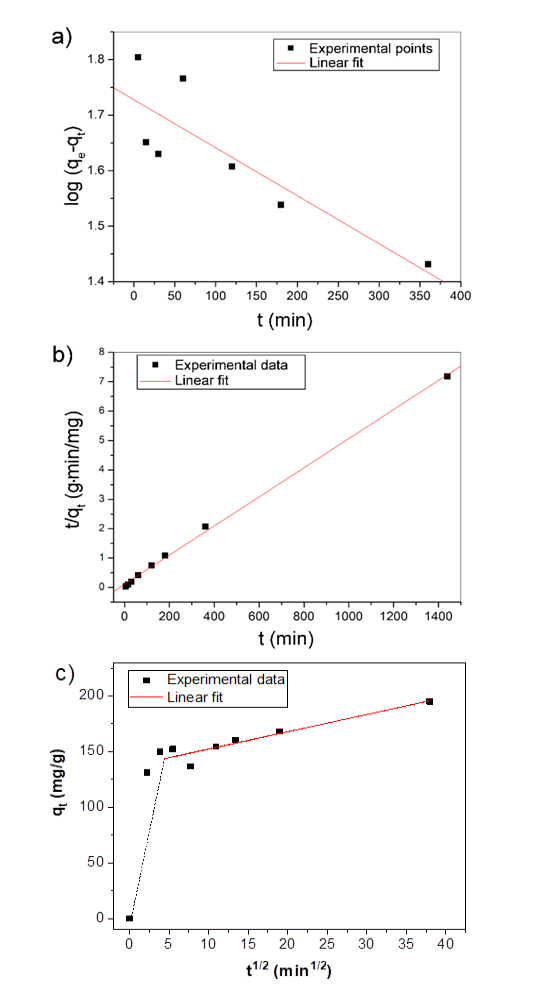


**Figure S1.** Linear fitting of the kinetic data for initial Sr concentration of 2800 mg/L using pseudo-first order (a) pseudo-second order model (b), and intraparticle diffusion model (c).

| Kinetic parameters |  | |
| --- | --- | --- |
| q_e(exp)_ (mg/g) | | 200.3 |
| **Pseudo-first order model** | | |
| *k_p1_* (1/min) | | 1.99 ∙ 10^-3^ |
| *q_e_* (mg/g) | | 50.1 |
| *R^2^* | | 0.739 |
| **Pseudo-second order model** | | |
| *k_p2_* (g/mg∙min) | | 2.17 ∙10^-4^ |
| *q_e_* (mg/g) | | 202.0 |
| *R^2^* | | 0.998 |
| *h* (mg/g∙min) | | 8.85 |
| **Intraparticle diffusion model** | |  |
| *K_id_* (mg/g^.^min^1/2^) | | 1.465 |
| *C* (mg/g) | | 138.9 |
| *R^2^* | | 0.875 |

**Table S2.** Kinetic parameters of Sr sorption by Z4A (initial Sr concentration 2800 mg/L).


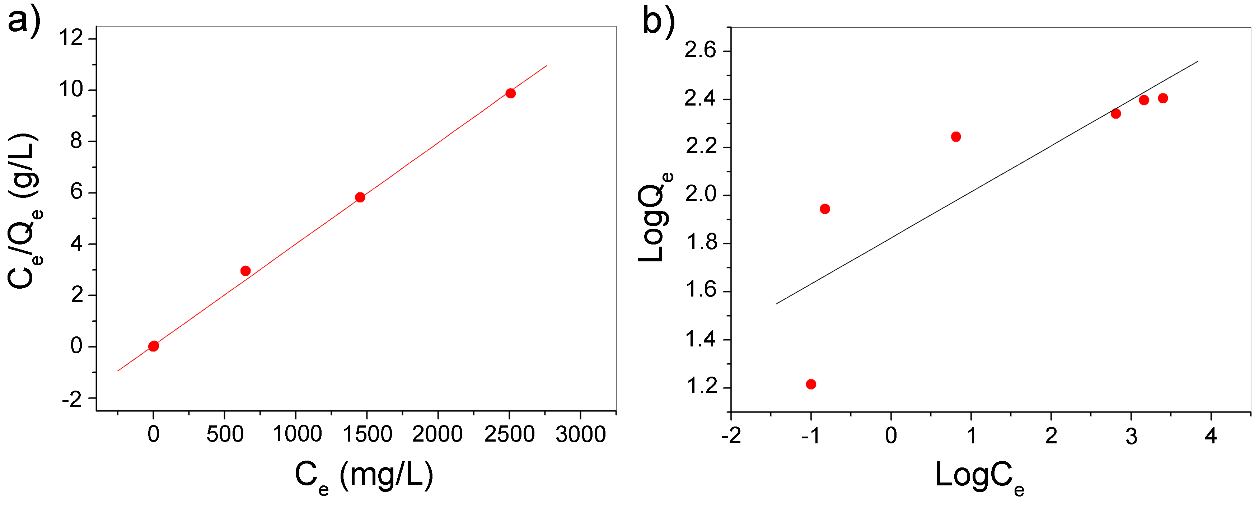


**Figure S2.** Fitting of equilibrium Sr sorption data by Z4A using the Langmuir (a) and Freundlich (b) model.


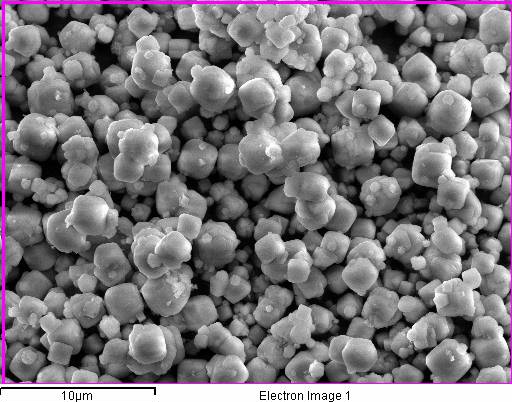
**
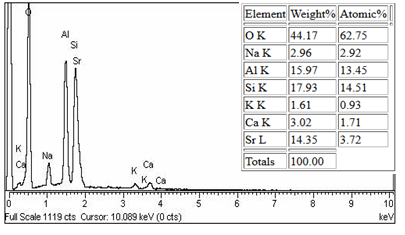
**

**Figure S3.** SEM/EDS analysis of Z4A sample after interaction with equimolar Sr, Ca, Mg, Na, and K mixture (0.02 mol/L of each cation).

| Synthetic seawater properties | | Groundwater properties | |
| --- | --- | --- | --- |
| pH | 8.3 | pH | 7.11 |
| Ca^2+^ | 440 mg/L | Ca^2+^ | 100.4 mg/L |
| Mg^2+^ | 1310 mg/L | Mg^2+^ | 21.2 mg/L |
| K^+^ | 400 mg/L | K^+^ | 2.08 mg/L |
| Na^+^ | 10500 mg/L | Na^+^ | 15.04 mg/L |
| Sr^2+^ | 8.25 mg/L | Sr^2+^ | 0.1 mg/L |
| SO_4_^2-^ | 2700 mg/L | SO_4_^2-^ | 27.2 mgL |
| Cl^-^ | 19450 mg/L | Cl- | 25.28 mg/L |
| Br^-^ | 56 mg/L | F^-^ | 74 µg/L |
| CO_3_^2-^/HCO_3_^-^ | 142 mg/L | NO_3_^-^ | 14.08 mg/L |
| I^-^ | 0.06 mg/L | Fe | < 20 µg/L |
| Salinity | 33 g/kg | Conductivity | 638.4 µS/cm |

**Table S3.** Properties of the synthetic seawater and the sample of groundwater used in sorption and desorption experiments, respectively

**References**

1. Lagergren, S. Zur theorie der sogenannten adsorption gelöster stoffe, Kungliga Svenska Vetenskapsakademiens. *Handlingar* **24**, 1–39 (1898).

2. Ho, Y. & McKay, G. Pseudo-second order model for sorption processes. *Process Biochem.* **34**, 451–465 (1999).

3. Weber, W.J. & Morris J.C. Kinetics of adsorption carbon from solutions. *J Sanit. Engeering Div. – Proc. Am. Soc. Civ. Eng*. **89**, 31–59 (1963).
